# Development and Clinical Application of a Novel Autonomic Transient Response-Based Screening System for Major Depressive Disorder Using a Fingertip Photoplethysmographic Sensor

**DOI:** 10.3389/fbioe.2018.00064

**Published:** 2018-05-22

**Authors:** Sumiyakhand Dagdanpurev, Guanghao Sun, Toshikazu Shinba, Mai Kobayashi, Nobutoshi Kariya, Lodoiravsal Choimaa, Suvdaa Batsuuri, Seokjin Kim, Satoshi Suzuki, Takemi Matsui

**Affiliations:** ^1^Graduate School of System Design, Tokyo Metropolitan University, Tokyo, Japan; ^2^Machine Intelligence Laboratory, School of Engineering and Applied Sciences, National University of Mongolia, Ulaanbaatar, Mongolia; ^3^Graduate School of Informatics and Engineering, The University of Electro-Communications, Tokyo, Japan; ^4^Department of Psychiatry, Shizuoka Saiseikai General Hospital, Shizuoka, Japan; ^5^Maynds Tower Mental Clinic, Tokyo, Japan; ^6^Department of Mechanical Engineering, Kansai University, Osaka, Japan

**Keywords:** heart rate variability, transient autonomic responses, mental task, major depressive disorder, photoplethysmographic sensor, logistic regression analysis

## Abstract

Over 350 million people across the world suffer from major depressive disorder (MDD). More than 10% of MDD patients have suicide intent, while it has been reported that more than 40% patients did not consult their doctors for MDD. In order to increase consultation rate of potential MDD patients, we developed a novel MDD screening system which can be used at home without help of health-care professionals. Using a fingertip photoplethysmograph (PPG) sensor as a substitute of electrocardiograph (ECG), the system discriminates MDD patients from healthy subjects using autonomic nerve transient responses induced by a mental task (random number generation) via logistic regression analysis. The nine logistic regression variables are averages of heart rate (HR), high frequency (HF) component of heart rate variability (HRV), and the low frequency (LF)/HF ratio of HRV before, during, and after the mental task. We conducted a clinical test of the proposed system. Participants were 6 MDD patients (4 females and 2 males, aged 23–60 years) from Shizuoka Saiseikai General Hospital psychiatry outpatient unit and 14 healthy volunteers from University of Electro-Communications (6 females and 8 males, aged 21–63 years). The average PPG- and ECG (as a reference)-derived HR, HF and LF/HF were significantly correlated with each other (HR; *r* = 1.00, *p* < 0.0001, HF; *r* = 0.98, *p* < 0.0001, LF/HF; *r* = 0.98, *p* < 0.0001). Leave-one-out cross validation (LOOCV) revealed 83% sensitivity and 93% specificity. The proposed system appears promising for future MDD self-screening at home and are expected to encourage psychiatric visits for potential MDD patients.

## Introduction

The number of patients diagnosed with major depressive disorder (MDD) has been increasing in recent years around the world and scientists predict that mood disorders induced by MDD will be the primary cause of mental disability by 2020 (Shinba, 2014; WHO, 2017). MDD has a variety of causes, including mental stress, which can lead neurogenic shock, cardiovascular diseases, neuropsychiatric diseases, gastrointestinal diseases, and increased suicidal intent (Selye, 1976; Kerr, 2015).

**Figure 1:**
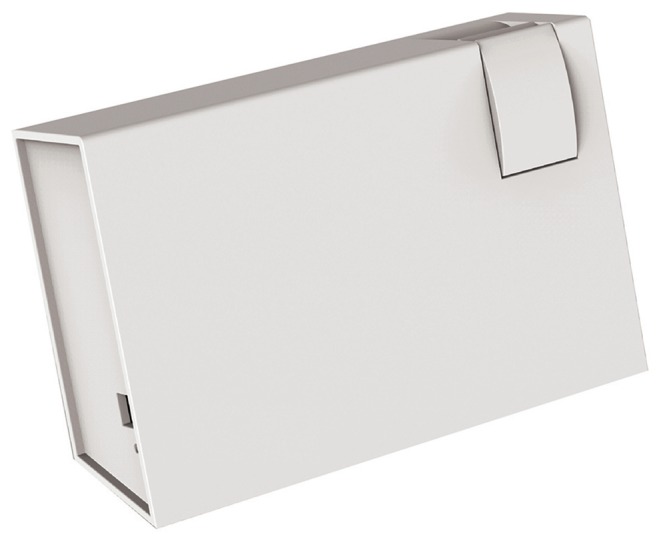
Main body of the novel screening system for major depressive disorder. The system uses a photoplethysmographic sensor to record response-based transient autonomic nerve activity.

Diagnostic systems for a variety of diseases are continually being improved and broadened. For instance, we have developed various infection screening systems using infrared cameras and microwave radars (Matsui et al., 2004, 2010; Sun et al., 2014; Yao et al., 2016). However, for people who might have MDD, going to psychiatric facilities with experienced doctors is laborious and stressful (Westen, 2012; Culpepper et al., 2015). Moreover, delaying the start of medical treatment can lead to exacerbated symptoms and greater daily disability. To overcome such incidents, we have developed an MDD screening method based on autonomic responses during a mental task, which combines heart rate variability (HRV) with logistic regression analysis. In our previous study, HRV was determined using electrocardiography (ECG). However, ECG measurement requires electrodes to be attached to a person's body, supervision of medical personnel, and inconveniences associated with any trips to a clinic or a hospital. To avoid such stressful and restrained screening for MDD, we have developed a novel MDD screening system that incorporates fingertip photoplethysmographic (PPG) sensor and logistic regression analysis (Figure 1).

A system which does not require medical personal help for electrode attachment can enable MDD screening at home or at the work place. Here we report a clinical test of the proposed MDD screening system that included 6 patients with MDD and 14 healthy control participants. The method was evaluated via leave-one-out cross validation (LOOCV).

## System design

### Heart rate variability as a tool for transient autonomic response measurement

In recent years, applicability of HRV power spectral analysis has gradually been extended from basic research to clinic practice, as evidenced by the large number of clinical studies that have been undertaken (Malik and Camm, 1995). Shinba reported that the HF component of the HRV, which corresponds to parasympathetic activation, differed between patients with MDD and healthy volunteers (Shinba, 2014). Compared with other techniques used to explore autonomic control, spectral analysis of HRV is unique because it provides an opportunity for a continuous examination of reciprocal changes in sympathetic and vagal modulation. According to HRV spectral analysis, most of the HRV signal power is concentrated in two frequency ranges: HF and LF. The HF band (0.15–0.4 Hz) is associated with parasympathetic activity and the LF band (0.04–0.15 Hz) is modulated by baroreflex-induced cardiac autonomic outflow (Goldstein et al., 2011). Based on these properties, our previous study confirmed that variance in HRV variables could be used as an MDD screening tool (Sun et al., 2016).

### Hardware design of the proposed system

**Figure 2:**
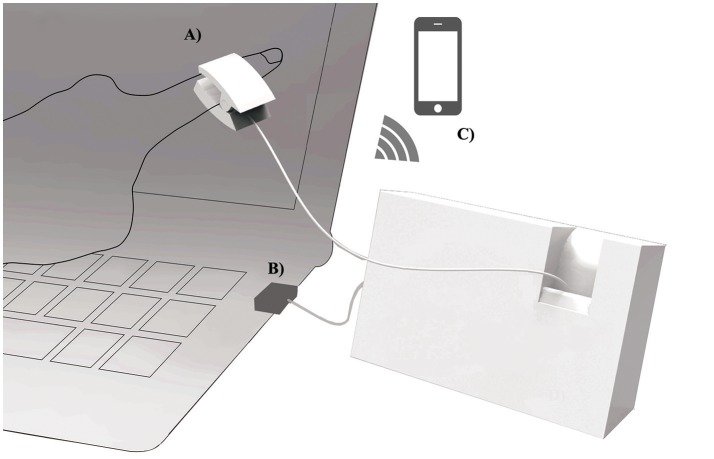
System configuration. **(A)** A photoplethysmographic sensor equipped finger clipper for pulse measurement. **(B)** USB connection to computer. **(C)** wireless connection to a smartphone.

The exterior design of the proposed system is shown in Figure 1. A PPG sensor was mounted on a finger clipper to measure fingertip pulse (Figure 2A). We used a PPG sensor with a greenish yellow light (wave length: 565 nm; Avago-technologies APDS-9008) and a 1-MHz low-power operation amplifier (MCP-6001). The amplifier output is transferred to a personal computer at a sampling rate of 100 Hz through a 12-bit AD converter with a USB port (Figure 2B). A wireless connection with a smartphone can be utilized as a graphic terminal (Figure 2C).

### Software design of the proposed system

The sampling and analyzing software was written in LabView graphical block diagram programming language (National Instruments, Texas, USA). The time series of peak to peak (PP) PPG intervals are interpolated using a polynomial and resampled at 4 Hz. This procedure was included to achieve equally spaced sampling for Fast Fourier Transform (FFT) and was necessary because PP intervals fluctuate with autonomic nerves activity. The power spectrum of resampled PP intervals was calculated using FFT every 2 s for 30 s of data. The power spectrum was then integrated from 0.04 to 0.15 Hz to calculate the LF component of the HRV and from 0.15 to 0.4 Hz to determine the HF component of the HRV. The logit of the logistic regression analysis that discriminates patients with MDD from healthy volunteers is determined by:

(1) $$\begin{array}{r} Logit\;score=\sum_{1\leq j\leq 3}^{1\leq i\leq 3}A_{ij}X_{ji}+constant \end{array}$$

The logistic regression coefficient matrix, *A*_*ij*_ and a *constant* are determined by logistic regression variables which were the mean HR, HF, and LF/HF for each time period (before, during, and after the mental task) for both groups of participants via machine learning software WEKA 3.8.1. *Xj1, Xj2*, and *Xj3* represent HR, HF, and LF/HF, respectively. The suffix *j* represents the three periods (1 = before, 2 = during, 3 = after). To precisely estimate screening accuracy, we conducted LOOCV, which is the preferred technique when estimating accuracy with a small data set. LOOCV is a particular case of *k*-fold cross-validation, where *k* equals the number of total instances. Thus, in each iteration a single observation is withheld from training, other recent samples are used for training, and the model is tested on that single observation after training is completed.

## Materials and methods

### Photoplethysmographic measurement

Fingertip PPG signal reflects arterial blood pulsation, which is modulated by autonomically influenced cardiac pulsation (Lin et al., 2013). We positioned the PPG sensor on the middle phalanx of an index finger. As a reference, the data derived by the proposed system was compared with that derived from ECG, and we calculated the Pearson correlation coefficients between the HR and HRV indices that were derived from the two different methods.

### Participants in the clinical test

Six patients with MDD (4 females and 2 males, aged 23–60 years) who were taking antidepressant medication were recruited from the psychiatry outpatient unit of Shizuoka Saiseikai General Hospital. Fourteen healthy volunteers (6 females and 8 males, aged 21–63 years) were recruited from the University of Electro-Communications. All participants provided written informed consent.

### Measurement protocol

**Figure 3:**
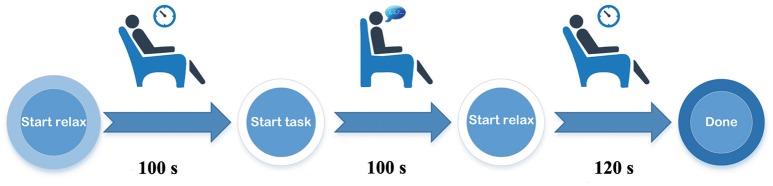
Schematic diagram of the measurement procedure.

Participants sat on a chair and had placed their fingers in the finger clip for PPG measurement. They chose a display device (computer monitor or a smartphone) that provided visual instructions. PPG sensors started to recording the HRV signals when the instruction began and continued for 320 s during the three periods (Figure 3) before, during, and after the mental task. Before the mental task (100 s), participants sat still and relaxed. During the mental task (100 s) they vocalized a random number from 0 to 9 each second. After mental task (120 s) they relaxed again.

## Results

**Figure 4:**
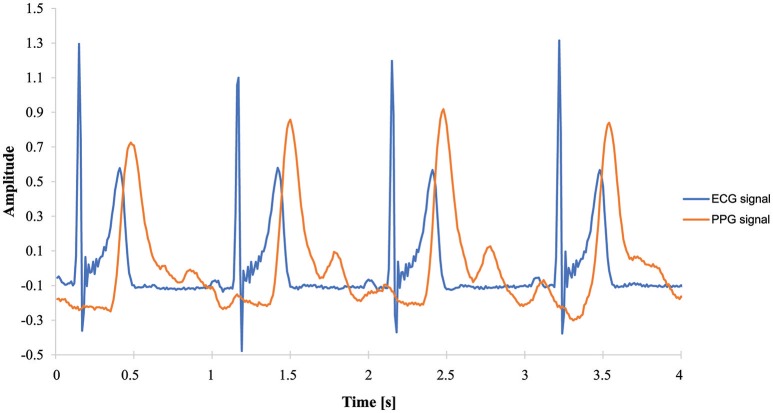
Amplitude over time, measured by ECG and PPG sensors.

Figure 4 shows the PPG and ECG amplitudes of a typical healthy volunteer for 4 s. The PPG peaked at approximately 300 ms after R wave was seen on the ECG. The PP intervals for PPG were almost the same as the RR intervals from the ECG.

**Figure 5:**
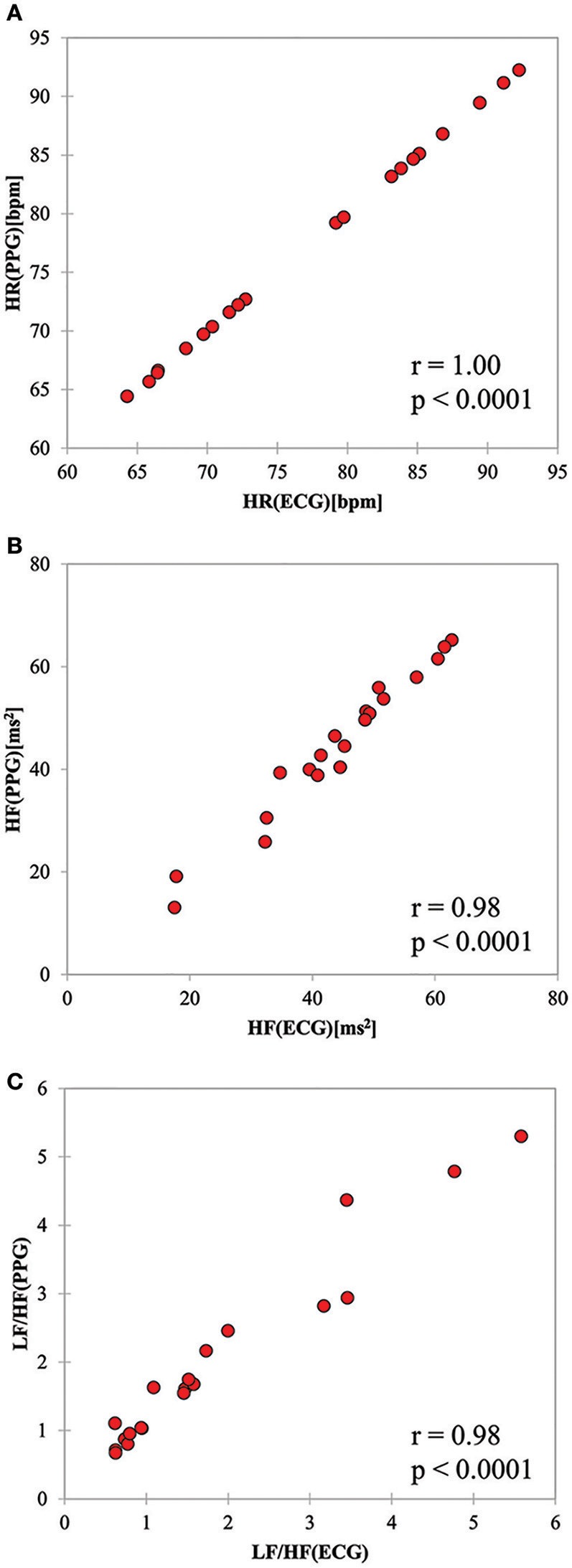
Scatter plots for **(A)** HR, **(B)** HF, and **(C)** LF/HF values derived from ECG and PPG sensors.

The HR, HF, and LF/HF determined by PPG significantly correlated with those calculated from ECG signals (HR; *r* = 1.0, *p* < 0.0001, HF; *r* = 0.98, *p* < 0.0001; LF/HF; *r* = 0.98, *p* < 0.0001). As shown in Figure 5, PPG- and ECG-derived HR were exactly linearly correlated, while HF and LF/HF were slightly less correlated. This can be explained by small artifacts in the PP intervals that affect frequency domain parameters such as HF and LF/HF.

**Figure 6:**
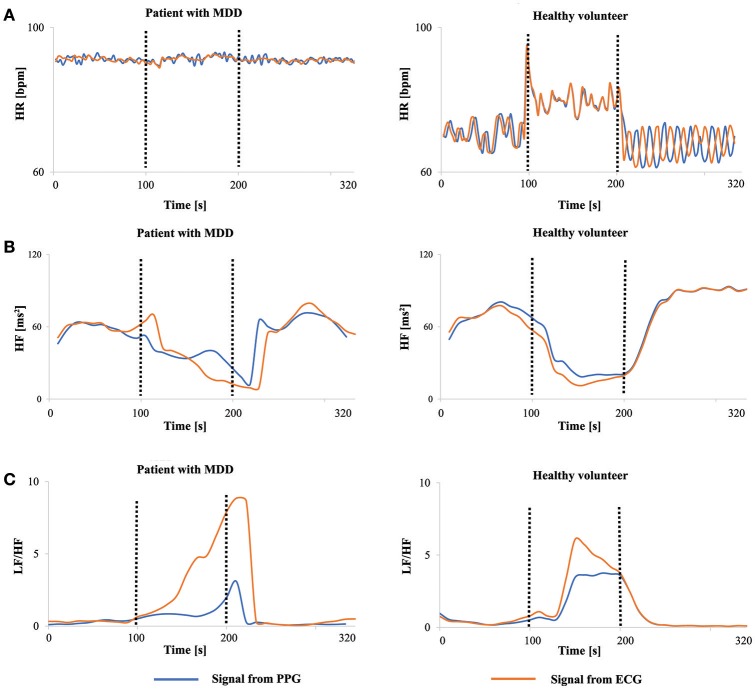
HRV and HR values before (0–100 s), during (100–200 s), and after (200–320 s) a mental task. Signals on the right are from a healthy volunteer and signals on left are from a patient. **(A)** HR-value. **(B)** HF-value. **(C)** LF/HF-value.

Figure 6 shows the PPG- and ECG-derived HR, HF, and LF/HF values for a typical healthy volunteer and a patient with MDD during the experiment. The horizontal axis spans one before/during/after cycle of the task (320 s). The HR, HF and LF/HF values for the healthy volunteer showed a drastic change during the mental task. In contrast, the HR of the patient did not change during the mental task, while the HF and LF/HF showed a later response to the mental task than what was observed in the healthy volunteer. PPG- and ECG-derived HR and HF match fairly well, while the task-induced response in LF/HF determined by ECG was higher than that derived by PPG.

The logit score that distinguishes patients with MDD from healthy volunteers was calculated by Equation (1). The multiple logistic regression coefficients A_ij_ and corresponding logistic regression variables X_ji_ in Equation (1) are listed in Table 1.

**Table 1:** Logistic regression variables and corresponding multiple logistic regression coefficients.

**Logistic regression variables**	**Corresponding multiple logistic regression coefficients**
HR Before MT(X_11_)	0.23 (A_11_)
HR During MT(X_21_)	6.43 (A_12_)
HR After MT(X_31_)	−9.7 (A_13_)
HF Before MT(X_12_)	−4.97 (A_21_)
HF During MT(X_22_)	4.73 (A_22_)
HF After MT(X_32_)	−3.59 (A_23_)
LF/HF Before MT(X_13_)	−36.78 (A_31_)
LF/HF During MT(X_23_)	30.81 (A_32_)
LF/HF After MT(X_33_)	−87.24 (A_33_)
	605.15 (constant)

**Figure 7:**
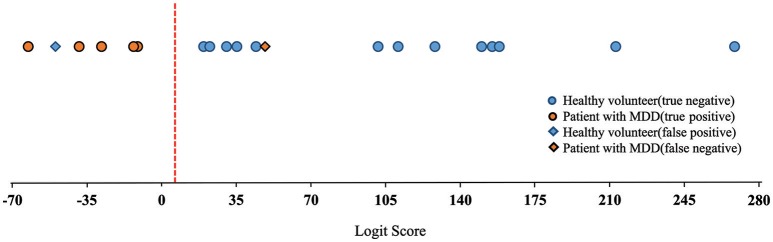
Discrimination results represented by plotting the logit score.

Equation (1), i.e., linear combination of nine variables which determine logit score indicated statistical significance (*p* < 0.01), and it achieved high screening sensitivity (83%) and specificity (93%) (Figure 7), although *F*-test and *t*-test (GraphPad Software) revealed that some variables are not statistically significant (HF_duringMT_, HF_afterMT_, LF/HF_beforeMT_, and LF/HF_duringMT_) as shown in Table 2.

**Table 2:** Paired *t*-test and *F*-test result between patients with MDD and healthy volunteers' heart rates (HR) and heart rate variability (HRV) indices.

**Variables**		**HR(bpm)**			**HF(ms**^**2**^**)**			**LF/HF**		
		**Before MT**	**During MT**	**After MT**	**Before MT**	**During MT**	**After MT**	**Before MT**	**During MT**	**After MT**
*p*-value		0.01	0.03	0.02	0.05	0.17	0.11	0.42	0.32	0.03
*F*-test value		1.03	1.11	0.93	0.33	4.82	0.44	1.65	5.98	0.14
Statistical significance level		**	*	*	*	*ns*	*ns*	*ns*	*ns*	*
Mean ±*SD*	MDD	84.7 ± 8.4	87.9 ± 8	82.2 ± 8.3	56.4 ± 54.4	29.4 ± 21.3	62.8 ± 31.8	3.5 ± 1.3	6.6 ± 2.8	4.6 ± 2.76
	Healthy	73.8 ± 8.5	77.8 ± 8.5	70 ± 8.3	365.9 ± 519.4	109.9 ± 170.8	334.4 ± 370.1	1.3 ± 2.5	5 ± 5.4	±1

Mean values with standard deviation (SD) of HR and HRV indices before, during and after mental task (MT). Statistical significance level:

**, very significant;

*, *significant; ns, not significant*.

Randomized quantile residuals analysis (Dunn and Smyth, 1996; Spyroglou et al., 2016) revealed that the logistic regression model is suitable for MDD patient classification. Furthermore, the Anderson- Darling test was performed with *A* = 0.23 and *P* = 0.77.

This clinical study had 20 participants, which was a small sample size compared with large scale clinical studies. Therefore, we adopted LOOCV analysis, which was appropriate for accurately estimating small datasets (Yao et al., 2016). The logit score could potentially be used as an index of depression or mental health severity because it indicates a positive correlation with the SDS score that was used to screen for MDD (Shinba, 2014). The center of gravity for the logit score for the healthy volunteers was greater than that for the patients with MDD by about 120.

## Discussion

The high rate of suicidal intentions associated with increasing numbers of patients with MDD motivated us to develop a self-monitoring system for MDD screening. Conventionally, MDD is diagnosed by subjective methods such as self-report screening questionnaires or psychiatrist interviews based on Diagnostic and Statistical Manual of Mental Disorders V (DSM-V) published by American Psychiatric Association. Our previous study (Sun et al., 2016) adopted mental task induced autonomic response determined by ECG-derived HRV for MDD screening. The ECG-derived HRV based method achieved 80% sensitivity and 79% specificity via 91 subjects' (44 patients with MDD and 47 normal volunteers) HRV dataset. However, ECG measurement is not applicable at home, school and workplaces and needs assistance of health-care professionals because it requires silver electrodes to be attached to body surface in order to reduce skin resistance. Therefore, comfort enhanced and user-friendly self-monitoring system for MDD screening is proposed in this paper. The system described here adopts a fingertip PPG sensor, which has several advantages over the initial ECG system that we previously described (Sun et al., 2016).

Proposed fingertip PPG sensor-based MDD screening system adopting multiple logistic regression model with LOOCV achieved 83% sensitivity and 93% specificity in distinguishing 6 patients with MDD from 14 healthy volunteers. In addition, the system appears promising not only for MDD screening but also for determining MDD severity levels.

The limitation of present study is that we tested the system with a rather small sample size of patients and healthy volunteers. Therefore, further study should involve more participants and be carried out to compare screening accuracy of MDD patients with and without anti-depressant medication. Moreover, it would be important to compare system sensitivity and specificity with those of recent MDD assessment tools such as Self-Rating Depression Scale (SDS), Hamilton Rating Scale for Depression (HAMD), Geriatric Depression Scale (GDS), and Beck Depression Inventory (BDI) for system accuracy investigation.

The preliminary results in this paper indicate our system to be a promising device for automated self-screening of people who potentially have MDD, either at home or at offices and without help of medical personnel.

## Ethics statement

The study was approved by Tokyo Metropolitan University ethics committee.

## Author contributions

SD, GS, and TM: designed the research and wrote the manuscript; SK and SS: contributed to exterior design and development of system; MK performed the experiments and analyzed the results; NK and TS: supervised psychiatric aspects and conceived idea; LC and SB: contributed mathematical and statistical methods. All authors reviewed the manuscript.

### Conflict of interest statement

The authors declare that the research was conducted in the absence of any commercial or financial relationships that could be construed as a potential conflict of interest.
